# Host immune response mediates changes in *cagA* copy number and virulence potential of *Helicobacter pylori*

**DOI:** 10.1080/19490976.2022.2044721

**Published:** 2022-03-15

**Authors:** Sungil Jang, Lori M. Hansen, Hanfu Su, Jay V. Solnick, Jeong-Heon Cha

**Affiliations:** a Department of Oral Biology, Oral Science Research Center, Department of Applied Life Science, The Graduate School, BK21 Four Project, Yonsei University College of Dentistry, Seoul, Republic of Korea; b Department of Oral Biochemistry, School of Dentistry, Jeonbuk National University, Jeonju, Republic of Korea; c Center for Immunology and Infectious Diseases; Departments of Medicine and of Microbiology and Immunology, School of Medicine; University of California Davis, Davis, CA, USA; d Affiliated Stomatology Hospital of Guangzhou Medical University, Guangdong Engineering Research Center of Oral Restoration and Reconstruction, Guangzhou Key Laboratory of Basic and Applied Research of Oral Regenerative Medicine, Guangzhou, Guangdong, China

**Keywords:** *Helicobacter pylori*, *cagA*, *cagY*, virulence, immune response

## Abstract

*Helicobacter pylori* is the major risk factor for gastric cancer. *H. pylori* harboring the type IV secretion system (T4SS) and its effector CagA encoded on the *cag* pathogenicity Island (*cag*PAI) increases the risk. *H. pylori* PMSS1 has a multi-*cagA* genotype, modulating *cagA* copy number dynamically from zero to four copies. To examine the effect of the immune response on *cagA* copy number change, we utilized a mouse model with different immune status. PMSS1 recovered from *Rag1^−/−^* mice, lacking functional T or B cells, retained more *cagA* copies. PMSS1 recovered from *Il10*^−/−^ mice, showing intense inflammation, had fewer *cagA* copies compared to those recovered from wild-type mice. Moreover, *cagA* copy number of PMSS1 recovered from wild-type and *Il10*^−/−^ mice was positively correlated with the capacity to induce IL-8 secretion at four weeks of infection. Since recombination in *cagY* influences T4SS function, including CagA translocation and IL-8 induction, we constructed a multiple linear regression model to predict *H. pylori*-induced IL-8 expression based on *cagA* copy number and *cagY* recombination status; *H. pylori* induces more IL-8 secretion when the strain has more *cagA* copies and intact *cagY*. This study shows that *H. pylori* PMSS1 in mice with less intense immune response possess higher *cagA* copy number than those infected in mice with more intense immune response and thus the multi-*cagA* genotype, along with *cagY* recombination, functions as an immune-sensitive regulator of *H. pylori* virulence.

## Introduction

*Helicobacter pylori* causes chronic gastritis and is the major risk factor for peptic ulcers, gastric adenocarcinoma, and mucosa-associated lymphoid tissue lymphoma.^1,2^ The fact that this bacterial species colonizes over half the human population,^3^ and gastric cancer is the third most frequent cause of cancer-related death,^4^ suggests the importance of *H. pylori* in human health. To achieve a successful lifelong colonization in the human stomach, the bacteria must adapt to highly variable host environments, which change with age and health conditions. In fact, genetic variation among *H. pylori* strains is sufficient to reflect genetic differences in human populations from which they were isolated.^5,6^ This suggests that *H. pylori* and human hosts have undergone coevolution, interacting with each other, which affects disease development.^7^ This *H. pylori* genomic evolution has been demonstrated not only in cross-sectional population studies, but also in real time during natural infection of humans, as well as experimental infection of humans and laboratory animals.^8–14^

It has long been reported that *H. pylori* strains harboring a genomic locus termed the *cag* pathogenicity Island (*cag*PAI) are associated with a higher risk of developing gastric adenocarcinoma.^15^ The *cag*PAI is a ~40-kb region containing 27-31 genes that encode a bacterial type IV secretion system (T4SS) and its effector protein, CagA,^16^ a 120 to 145-kDa protein that is injected into host gastric epithelial cells.^17^ Once inside the host cells, CagA is phosphorylated by host kinases^18,19^ and influences various host cellular processes in a phosphorylation-dependent and independent manner.^20,21^ CagA induces nuclear translocation of β-catenin,^22^ which induces the expression of genes involved in carcinogenesis, such as vascular endothelial growth factor, *NANOG, OCT4*, and *CDX1*.^23–25^ CagA also promotes survival and proliferation of gastric epithelial cells by activating ERK/MAPK and JAK/STAT3 pathways.^26–28^

CagA can be both beneficial and detrimental to the survival of *H. pylori* in the stomach. On the one hand, CagA induces loss of tight junctions and epithelial cell polarity by disrupting proper localization of junctional adhesion molecule and zona occludens-1,^29,30^ and inhibiting activity of partitioning-defective 1/microtubule affinity-regulating kinase.^31,32^ By disrupting epithelial cell polarity and modulating the transferrin-recycling pathway, CagA induces leakage of iron across the epithelial barrier, which is beneficial for growth and colonization of *H. pylori*.^33,34^ On the other hand, CagA mediates inflammation by inducing expression of pro-inflammatory cytokines in gastric epithelial cells.^35–37^ *In vitro*, CagA-expressing *H. pylori* strains induce higher levels of interleukin (IL) -8 secretion in gastric epithelial cells.^35^ CagA is also highly immunogenic, and seropositivity of antibody against CagA is associated with a higher degree of inflammation in stomach tissue,^38,39^ which is detrimental to survival and proliferation of *H. pylori*.^40,41^ Mongolian gerbils infected with *cagA*-deleted 7.13 *H. pylori* mutant strain showed increased colonization density and attenuated inflammation compared to those infected with 7.13 wild-type strain.^42^ CagA is associated with a Th1-polarized immune response,^43^ which mediates protection against *H. pylori* and clearance of infection.^44,45^ The beneficial and detrimental effects of CagA suggest that *H. pylori* needs to regulate CagA during colonization of *H. pylori* in the human stomach.

*H. pylori* has relatively few two-component regulatory systems, and instead often uses DNA recombination or slipped strand synthesis to regulate gene expression.^46–52^ For example, *H. pylori* modulates T4SS function, including CagA translocation and IL-8 induction, via recombination in *cagY*.^53^ CagY is an approximately 220-kDa protein that is an essential component of the T4SS core complex.^54–56^ *cagY* contains variable numbers of direct DNA repeats in its 5′ and middle regions, yielding variant CagY proteins by in-frame DNA recombination.^57,58^ Recombination in the middle repeat region of *cagY* was observed in *H. pylori* strains recovered from mice, gerbils, and monkeys.^53,59,60^ Typically, *cagY* recombination reduced T4SS function and decreased the host inflammatory response, although the recombination could occasionally increase T4SS function.^53,59,60^

Another potential DNA-based mechanism to regulate CagA expression is alteration of *cagA* gene copy number. About 7.5% of clinical *H. pylori* isolates in the United States carry a multi-*cagA* genotype, including strain PMSS1, which was shown to modulate *cagA* copy number dynamically from zero to four *cagA* copies *in vitro*.^61–63^ In *H. pylori* strains with multi-*cagA* genotype, *cagA* is flanked with ~0.5-kb direct repeats referred to as *cagA* homologous area (CHA)-ud and recombination between CHA-ud repeats is thought to be responsible for *cagA* copy number change.^62,63^ Among isogenic PMSS1 mutants with different *cagA* copy number, mutants with the higher *cagA* copy number expressed more CagA protein and translocated more CagA into gastric epithelial cell lines, which induced more cell elongation and IL-8 secretion,^62^ suggesting an association between *cagA* copy number and virulence.

To examine the association among changes in *cagA* copy number, virulence potential of *H. pylori*, and the intensity of host immune response, we measured *cagA* copy number in *H. pylori* strains isolated from experimental infection of wild-type, immunodeficient and hyper-immunosensitive mice. The results demonstrated an immune-sensitive change in the multi-*cagA* genotype. We conclude that modulating *cagA* gene copy number may play a role in regulating virulence during the colonization of *H. pylori* in stomach.

## Results

### *Host immune response mediates changes in* cagA *copy number*

**Figure 1.**
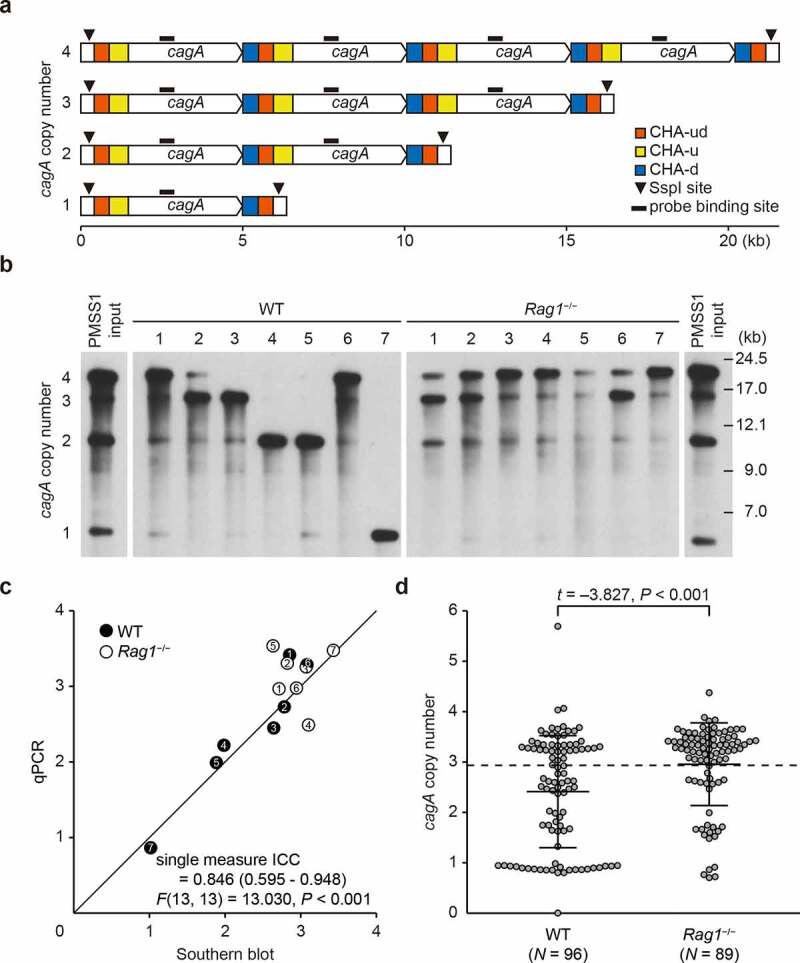
**Variation of *cagA* copy number in mice output *H. pylori* populations**. (a) Schematic diagram showing the principle of measuring *cagA* copy number in *H. pylori* strain PMSS1 by Southern blot. *cagA* gene and its flanking sequences [*cagA* homologous area (CHA)], SspI restriction sites, and probe binding sites were shown (modified from Jang, et al. 2017^62^). (b) *cagA* copy number of *H. pylori* populations isolated from 7 WT and 7 *Rag1*^−/−^ mice was analyzed by Southern blot. (c) The agreement between Southern blot and qPCR was evaluated by two-way mixed, single measure intraclass correlation coefficient (ICC). ICC was represented at the bottom of the plot with 95% confidence interval in parentheses. *F* statistic was represented with degrees of freedom in parentheses. Average *cagA* copy numbers of *H. pylori* populations isolated from each mouse were measured by densitometry analysis of Southern blot (horizontal axis) and those of 6 selected colonies were measured by qPCR (vertical axis). Each circle indicates a single *H. pylori* population and a number inside the circle indicates the mouse from which the population was isolated. Diagonal line indicates points where qPCR and Southern blot values are equal. (d) *cagA* copy number was measured in colonies isolated from WT and *Rag1*^−/−^ mice after 8 weeks from infection and described in a scatterplot. Each circle indicates individual colony. A dashed horizontal line on the plot background indicates mean *cagA* copy number of input population (2.93). Three lines in the scatterplot indicate mean ± standard deviations.

To investigate the effect of host immunity on *H. pylori cagA* copy number, C57BL/6 wild-type (WT) or *Rag1*^−/−^ mutant mice were infected with PMSS1 *H. pylori* for 8 weeks. After the infection period, the pattern of *cagA* copy number in *H. pylori* was examined by Southern blot using chromosomal DNA isolated from PMSS1 populations (sweeps) recovered from each mouse. As shown previously,^61,62^ input strain PMSS1 showed bands of 21.0 kbp, 15.9 kbp, 10.8 kbp, and 5.8 kbp; these sizes match those predicted for 4, 3, 2 and 1 copies of *cagA*, respectively (Figure 1(a), (b)). This suggests that strain PMSS1 consists of a heterogeneous population in terms of *cagA* copy number (Figure 1(b)). Interestingly, PMSS1 populations recovered from each WT or *Rag1*^−/−^ mouse showed different patterns of Southern blot, indicating various *cagA* copy numbers. Populations isolated from WT1 and WT6 mice had four *cagA* genes as their most common genotype; populations from WT2 and WT3 showed three *cagA* genes as the major genotype; WT4 and WT5 showed two *cagA* genes; and WT7 carried a single *cagA* gene (Figure 1(b), left panel). *H. pylori* populations isolated from *Rag1*^−/−^ 3, 4, and 7 mice showed four *cagA* genes as their most common *cagA* copy number; isolates from *Rag1*^−/−^ 1, 2, and 6 mice showed three *cagA* genes as the major *cagA* copy number; and strains from *Rag1*^−/−^ 5 mouse showed similar intensities of 3 bands for two, three, and four *cagA* copies (Figure 1(b), right panel). Although not all PMSS1 populations in each mouse showed the same patterns of *cagA* copy number, populations from WT mice contained more variable Southern blot profiles and decreased *cagA* copy number compared to those from *Rag1*^−/−^ mice. These results suggest that host immune response in mice affects *cagA* copy number in the colonizing *H. pylori* population.

To confirm these *cagA* copy number results, we performed densitometry analysis of the Southern blot profiles from each mouse. Next, we measured the *cagA* copy number of 6 randomly selected colonies from each mouse using qPCR as previously described.^62^ The average *cagA* copy number of the 6 selected colonies was compared to the *cagA* copy number of the *H. pylori* population calculated by densitometry analysis of Southern blot profiles. The agreement between the two measurements was evaluated by a two-way mixed, single measure intraclass correlation coefficient (ICC). ICC showed a good absolute agreement between Southern blot and qPCR analysis (ICC = 0.846, *P* < .001, Figure 1(c)), suggesting that the selected six colonies could effectively represent the mouse output *H. pylori* population from which they were isolated. Considering the heterogeneity of *H. pylori* sweeps, analysis of single colonies is required to further examine the changes in *cagA* copy number and its role in virulence. Thus, approximately six *H. pylori* colonies from each mouse were used for subsequent quantitative analyses.

Using this single colony approach, we first compared average *cagA* copy number in 5–6 colonies isolated from each of 16 C57BL/6 WT (*N* = 96) and 15 *Rag1*^−/−^ mice (*N* = 89) and compared the results to input strain PMSS1. WT mice output colonies carried an average of 2.41 (± 1.11) copies of *cagA*, while *Rag1*^−/−^ mice-output colonies had 2.96 (± 0.82) *cagA* copies, similar to input PMSS1, which was 2.93 (± 0.07) (Figure 1(d)). Average *cagA* copy number of output colonies from WT mice was significantly lower than that of the input population (*P* < .001, one-sample *t*-test for lower tail), while *Rag1*^−/−^ output colonies carried similar number of *cagA* copies to the input population (*P* = .799, one-sample two-tailed *t*-test). Average *cagA* copy number was higher in *Rag1*^−/−^ output colonies than WT mice output colonies (*P* < .001). These results suggest that the immune response in WT mice selects for a reduced *cagA* copy number, which is unaffected in *Rag1*^−/−^ mice.

### *Effect of host immune response on association among* cagA *copy number change*, cagY *recombination, and IL-8 induction*

**Figure 2.**
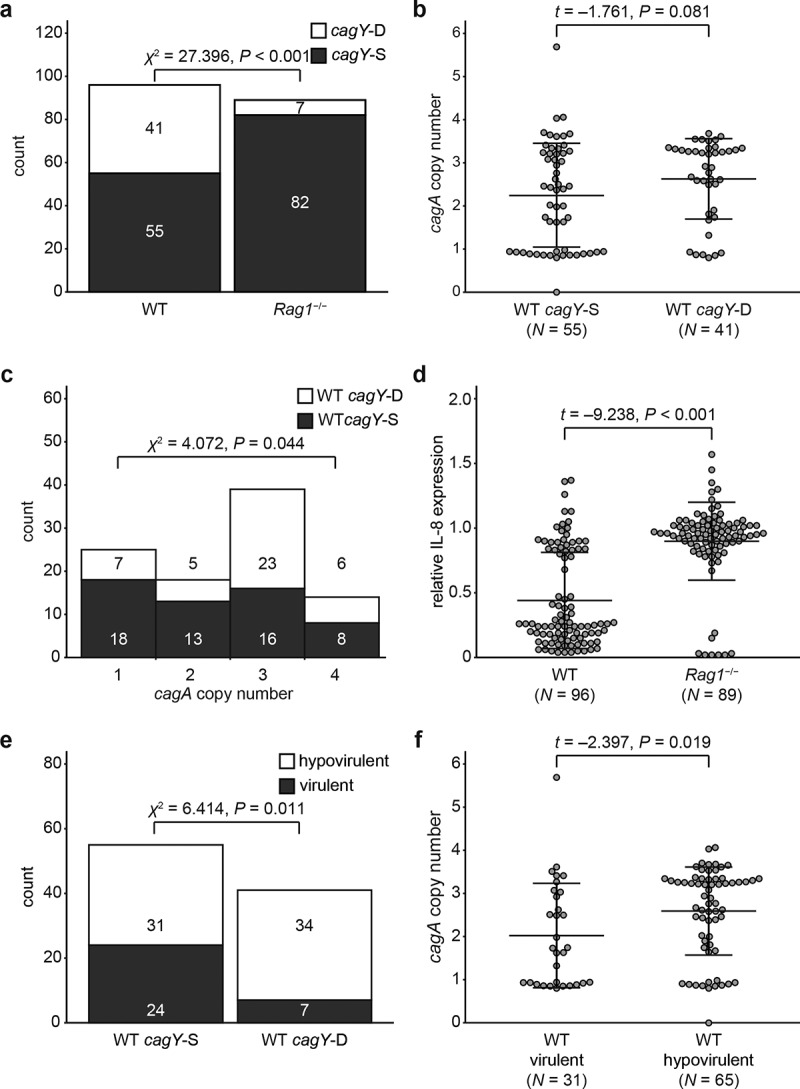
**Effect of host immune response on IL-8 induction and *cagA* copy number**. (a) Colonies recovered from WT or *Rag1*^−/−^ mice 8 weeks PI were classified according to *cagY* recombination status and number of colonies was indicated. (b) *cagA* copy number was measured in *cagY*-S and *cagY*-D colonies isolated from WT mice 8 weeks PI. (c) WT mice-output colonies were classified according to their *cagA* copy number and *cagY* recombination status, and the number of colonies in each group was described. Group 1: 0 ≤ *cagA* copy number < 1.5; Group 2: 1.5 ≤ *cagA* copy number < 2.5; Group 3: 2.5 ≤ *cagA* copy number < 3.5; Group 4: 3.5 ≤ *cagA* copy number. Association between *cagY* recombination and *cagA* copy number was examined using chi-square test for trend in proportions.(d) Induction of IL-8 was measured in colonies isolated from WT and *Rag1*^−/−^ mice and described in a scatterplot. (e) WT mice-output colonies were classified according to their *cagY* recombination status and virulence potential, and the number of colonies in each group was described. A colony was classified to be ‘hypovirulent’ if it induced less IL-8 than half of that induced by input strain PMSS1, otherwise classified to be ‘virulent’. (f) *cagA* copy number was measured in ‘virulent’ and ‘hypovirulent’ WT-output colonies. Each circle in scatterplots (panels b, d, and f) indicates individual colony. Three lines in the scatterplot indicates mean ± standard deviations.

It has been reported that the host immune response induces variation of CagY function, which is caused by recombination in repeat regions of *cagY*.^53,57,59^ This variation of CagY can alter T4SS function, which typically reduces virulence potential that is often evaluated by measuring the induction of IL-8 secretion.^53,59^ Therefore, we next examined the association between *cagA* copy number and *cagY* recombination in WT and *Rag1*^−/−^ mice output colonies. Recombination of *cagY* was determined by *cagY* PCR-RFLP pattern as described previously.^53,59^ Based on the PCR-RFLP pattern, *cagY* in mouse output colonies was classified as same (*cagY*-S) or different (*cagY*-D), compared with that of the input strain. RFLP result of input strain and representative RFLP results of *cagY*-S and *cagY*-D colonies are shown in Figure S1. Among 96 colonies recovered from WT mice, 41 (42.7%) were *cagY*-D, significantly higher than only 7 of 89 (7.9%) recovered from *Rag1*^−/−^ mice (*P* < .001, Figure 2(a)). This difference in the distribution of *cagY*-S and -D is consistent with previous results.^59^ Next, to examine how *cagY* recombination and *cagA* copy number are associated, we compared *cagA* copy number of strains according to whether they were *cagY*-S or *cagY*-D in strains from WT mice that yielded sufficient number of both *cagY*-S and -D colonies. Average *cagA* copy number of *cagY*-S and *cagY*-D colonies in WT mice was 2.24 (± 1.21) and 2.63 (± 0.93), respectively (Figure 2(b)). *cagY*-S colonies carried fewer *cagA* copies than *cagY*-D colonies, though the *P*-value was slightly above the significance threshold (*P* = .081). On the other hand, WT-output colonies carrying more *cagA* copies were likely to have recombined *cagY* (*P* = .044, Figure 2(c)).

The function of the *H. pylori* T4SS is governed not only by *cagY* recombination but also by other genes located inside or outside *cag*PAI.^64–70^ Thus, *cagY*-S strains may have a nonfunctional T4SS, and *cagY*-D strains may have a functional T4SS, as reported previously.^53^ Secretion of IL-8 from *H. pylori*-infected cells has been used to measure the capacity of *H. pylori* to induce host inflammation. We therefore measured the relative amount of IL-8 secretion induced by each single colony compared to that induced by the input strain and used it to evaluate the virulence potential of each single colony isolate. As a result, average IL-8 induction of WT output colonies [0.44 (± 0.37)] was lower than that of *Rag1*^−/−^ output colonies [0.90 (± 0.30)] (*P* < .001, Figure 2(d)). Since relative induction of IL-8 showed a bimodal distribution centered around 0.5, we classified strains inducing IL-8 less than half of that induced by input strain PMSS1 as ‘hypovirulent’ and the rest as ‘virulent’. Among WT output colonies, 65 (67.7%) were ‘hypovirulent’, while only 8 colonies (9.0%) isolated from *Rag1*^−/−^ mice were ‘hypovirulent’. Among 96 colonies isolated from WT mice, 31 out of 55 *cagY*-S colonies (56.4%) nevertheless were ‘hypovirulent’, while 34 out of 41 (82.9%) *cagY*-D colonies were ‘hypovirulent’ (Figure 2(e)). Though there was an association between recombination of *cagY* and low virulence potential (*P* = .011, Figure 2(e)), our results imply that *cagY* recombination was not the sole cause of reduction of virulence potential, as recently reported.^65^ Strains classified as ‘virulent’ carried fewer *cagA* copies than ‘hypovirulent’ strains (*P* = .019, Figure 2(f)).

### *Stronger immune response is associated with lower* cagA *copy number*

**Figure 3.**
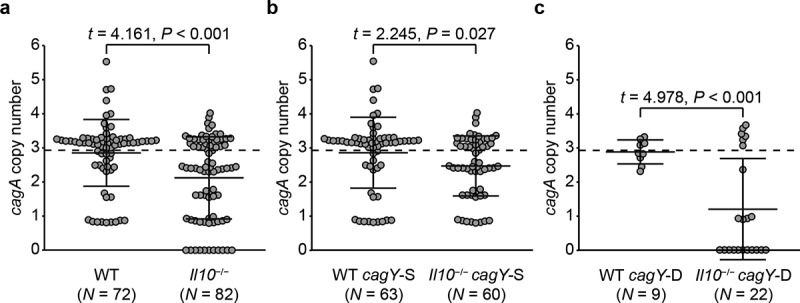
**Changes of *cagA* copy number in WT and *Il10*^−/−/−^ mice-derived*H. pylori* colonies according to *cagY* recombination status**. *cagA* copy number was measured in colonies isolated from WT and *Il10*^−/−/−^ mice 4 weeks PI. Colonies were further classified according to *cagY* recombination status. *cagA* copy number of each colony was described in a scatterplot. (a) All colonies, (b) *cagY*-S colonies, and (c) *cagY*-D colonies. Each circle indicates an individual colony. A dashed horizontal line on the plot background indicates mean *cagA* copy number of input population (2.93). Three lines in the scatterplot indicate mean ± standard deviations.

Since a functional immune response is necessary to select for reduced *cagA* copy number, we next asked whether the intensity of the immune response is associated with *cagA* copy number reduction. It has been reported that *H. pylori* represses immune response by inducing expression of IL-10, an anti-inflammatory cytokine,^71–73^ and therefore mice deficient with IL-10 (*Il10*^−/−/−^) show a more intense immune response against *H. pylori* than WT mice.^74^ Thus, we analyzed the *cagA* copy number in colonies isolated from WT and *Il10*^−/−/−^ mice, which were previously demonstrated to have markedly increased gastric inflammation.^59^ Because the *H. pylori* load in *Il10*^−/−/−^ mice rapidly decreases as the infection duration prolongs,^59^ the experimental period was shortened to 4 weeks to obtain sufficient number of colonies for examination. This enables evaluating the effect of intense inflammatory response on *cagA* copy number change, while data obtained from *Rag1*^−/−^^/−^ and *Il10*^−/−/−^ mice cannot be directly compared due to the difference in experiment duration. At 4 weeks post-infection (PI), 72 colonies were isolated from 12 WT mice, and 82 colonies were isolated from 15 *Il10*^−/−/−^ mice. Colonies from *Il10*^−/−/−^ mice carried an average of 2.12 (± 1.20) copies of *cagA*, which was significantly fewer than colonies from WT mice (2.85 ± 0.98, *P* < .001, Figure 3(a)). In 11 colonies from 2 *Il10*^−/−/−^ mice, *cagA* was undetectable. These results suggest that *cagA* copy number tends to decrease as the intensity of host immune response increases.

Next, to examine whether the recombination in *cagY* is associated with change of *cagA* copy number in an intense inflammatory environment, we analyzed *cagA* copy number of *H. pylori* colonies isolated from WT and *Il10*^−/−/−^ mice separately according to the recombination status in *cagY*. Among WT output colonies, 9 of 72 (12.5%) were classified as *cagY*-D, compared to 22 of 82 (26.8%) from *Il10*^−/−/−^ mice. When *cagY*-S output colonies of WT and *Il10*^−/−^^/−^ mice were compared, *Il10*^−/−^^/−^ mice output colonies had fewer *cagA* copies [2.46 (± 0.88)] than WT output colonies [2.85 (± 1.04)] (*P* = .027, Figure 3(b)). More drastic decrease in *cagA* copy number was observed in *cagY*-D colonies isolated from *Il10*^−/−/−^ mice. Average *cagA* copy number of *cagY*-D colonies isolated from *Il10*^−/−^^/−^ mice was 1.20 (± 1.48), while that of *cagY*-D colonies isolated from WT mice was 2.88 (± 0.35) (*P* < .001, Figure 3(c)). All colonies that lost all *cagA* genes were classified as *cagY*-D. Overall, both *cagY*-S and *cagY*-D colonies showed a tendency to have fewer *cagA* copies in a more intense inflammatory environment. However, in colonies that underwent recombination in *cagY*, change of *cagA* copy number might be more intensively affected by the intensity of inflammation.

### *Association of* cagA *copy number with IL-8 induction*

**Figure 4.**
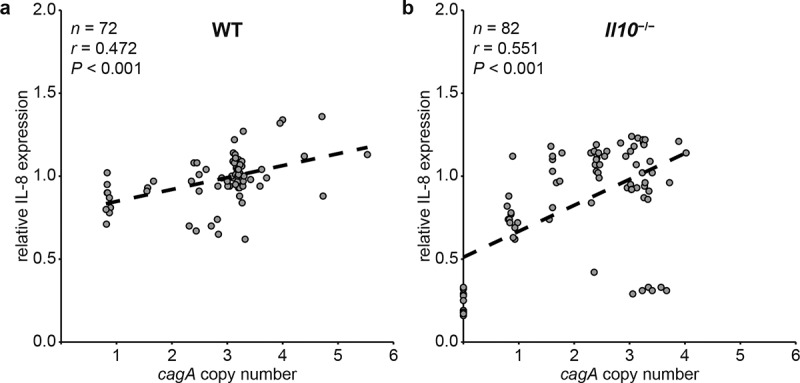
**Correlation between *cagA* copy number and induction of IL-8 secretion in AGS cells was measured in WT and *Il10*^−/−/−^ mice-derived*H. pylori* colonies**. Correlation between *cagA* copy number and induction of IL-8 secretion in AGS cells was analyzed by calculating Pearson correlation coefficient. *cagA* copy number and induction of IL-8 secretion of each colony were plotted. Each circle indicates individual colony. (a) WT mice-output colonies, and (b) *Il10*^−/−/−^ mice-output colonies.

Since PMSS1 strains carrying more *cagA* copies induce more IL-8 secretion *in vitro*,^62^ we next asked whether the association between *cagA* copy number and IL-8 induction would be maintained during *in vivo* mouse infection in a situation where recombination in *cagY* and change of *cagA* copy number are allowed to occur. To address this, we measured induction of IL-8 in 4-week mouse output colonies from WT and *Il10*^−/−/−^ mice and analyzed the correlation between *cagA* copy number and secretion of IL-8. We found a positive correlation between *cagA* copy number and IL-8 secretion in 72 colonies isolated from WT mice (*r* = 0.472, *P* < .001, Figure 4(a)), but the relationship held only among colonies in which *cagY* did not undergo recombination (*r* = 0.586, *P* < .001 for *cagY*-S colonies and *r* = 0.500, *P* = .171 for *cagY*-D colonies, Figure S2 a,b). A positive correlation between *cagA* copy number and IL-8 was also observed in 82 colonies isolated from *Il10*^−/−/−^ mice (*r* = 0.551, *P* < .001, Figure 4(b)). Similar to the WT output colonies, correlation between *cagA* copy number and IL-8 secretion was maintained only in *cagY*-S colonies (*r* = 0.469, *P* < .001 for *cagY*-S colonies and *r* = 0.160, *P* = .477 for *cagY*-D colonies, Figure S2 c,d). These results demonstrate that *cagA* copy number and capacity to induce IL-8 are positively correlated *in vivo*, a relationship that is driven primarily by colonies that have not undergone *cagY* recombination.

In *Rag1*^−/−/−^ mice-output colonies 8 weeks PI, the induction level of IL-8 secretion was hardly correlated with *cagA* copy number (Figure S3a), and the biased distribution of colonies to *cagY*-S made it problematic to compare *cagY*-S and *cagY*-D colonies (Figure S3b,c). In colonies isolated from WT mice, the more dispersed distribution of relative IL-8 expression resulted in a weak negative correlation between *cagA* copy number and IL-8 secretion (Figure S3d). A similar correlation pattern was observed when *cagY*-D colonies were examined while no correlation pattern was observed in *cagY*-S colonies (Figure S3e,f). These results suggest that unrevealed changes occurred in *H. pylori* during prolonged infection may lower the impact of *cagA* copy number on virulence.

### *Modeling the association of* cagA *copy number*, cagY *recombination, and IL-8 induction*

**Figure 5.**
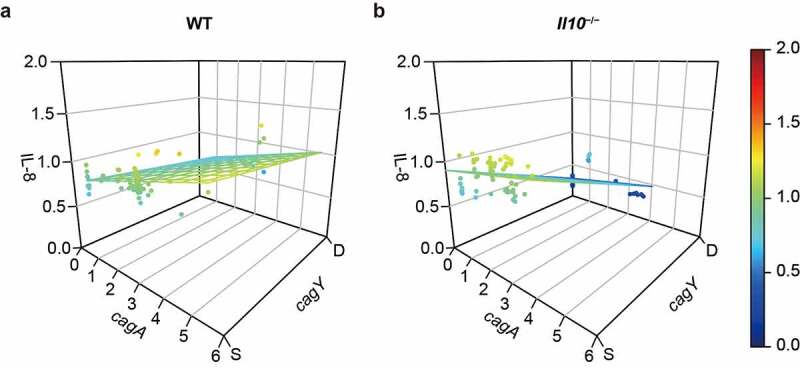
**Effect of *cagA* copy number and *cagY* recombination on IL-8 secretion in AGS cells was measured in WT and *Il10*^−/−/−^ mice-derived*H. pylori* colonies**. Induction of IL-8 secretion was predicted by multiple linear regression, based on *cagA* copy number and *cagY* recombination status of each mouse output colony. 3D scatterplot describing *cagA* copy number, *cagY* recombination status, and induction of IL-8 secretion of colonies isolated from (a) WT mice, and (b) *Il10*^−/−/−^ mice. Each circle indicates individual colony and colored plane indicates a regression plane.

Since both *cagY* recombination and *cagA* copy number affect the capacity to induce IL-8 in mouse output colonies, we sought to model the association by employing multiple linear regression (Figure 5, Table 1). The overall model suggests that relative IL-8 secretion will increase 0.073 units per 1 copy increase of *cagA* gene and decrease 0.189 units with *cagY* recombination in WT output colonies (R^2^ = 0.383, *P* < .001, Figure 5(a), Table 1, left panel). Similar but more robust relationships were observed in colonies isolated from *Il10*^−/−^^/−^ mice (R^2^ = 0.818, *P* < .001, Figure 5(b), Table 1, right panel). This analysis demonstrates that change in *cagA* copy number and recombination in *cagY* each contribute to the capacity to induce IL-8 in mouse output colonies, particularly in colonies from *Il10*^−/−^^/−^ mice.

**Table 1.** Effect of *cagA* copy number and *cagY* recombination on induction of IL-8 secretion in AGS cells, in 4-week mice output colonies

	4-week output colonies									
	WT (*N* = 72)					*Il10*^−/−^ (*N* = 82)				
predictors	B	SE	β	*t*	*P*	B	SE	β	*t*	*P*
*cagA* copy number	0.073	0.014	0.476	5.104	**< 0.001**	0.048	0.015	0.171	3.180	**0.002**
*cagY* recombination (*cagY*-S = 0, *cagY*-D = 1)	−0.189	0.042	−0.422	−4.522	**< 0.001**	−0.626	0.041	−0.815	−15.190	**< 0.001**
constant	0.798	0.043		18.506	< 0.001	0.908	0.042		21.643	< 0.001
	adjusted R^2^ = 0.383, *F* = 23.028, ***P* < .001**					adjusted R^2^ = 0.818, *F* = 182.741, ***P* < .001**				

B: unstandardized coefficient; SE: standard error of unstandardized coefficient; β: standardized coefficient. Boldface numbers indicate *P* values less than 0.05.

## Discussion

Previous studies reported that multi-*cagA* genotype was found in about 7.5% of clinical *H. pylori* isolates in the United States.^62,63^ *H. pylori* strain PMSS1, possessing multi-*cagA* genotype, was shown to consist of organisms with varying number of *cagA* genes, ranging from zero to four copies *in vitro*.^61,62^ Strains with more *cagA* copies expressed more CagA protein and translocated more CagA into gastric epithelial cell lines, which induced more cell elongation and IL-8 secretion.^62^ These results suggest an association between *cagA* copy number and virulence. Furthermore, increase of *cagA* copy number up to seven copies from single *cagA* was reported in a recent experimental *H. pylori* infection study on human subjects, which was conducted to examine bacterial adaptation during early stages of infection.^14^ Among the reisolates, some showed loss of ability to induce IL-8 secretion, which was assumed to be due to a mutation in *cagE* or *cagY*. Nonsynonymous mutations and sequence variations in genes encoding other *cag*PAI proteins or outer membrane proteins were also discovered in other reisolates. This implies that, during colonization of a new host, virulence potential of *H. pylori* may be altered by various factors including change in *cagA* copy number. Here, we have further investigated the immunologic basis and functional significance for the change in *cagA* copy number in multi-*cagA* genotype during *H. pylori* infection of mice.

Our results suggest that the host immune response affects *cagA* copy number. *Rag1*^−/−^^/−^mice-output colonies carried more *cagA* copies than WT mice-output colonies, and *Il10*^−/−^^/−^ mice-output colonies carried fewer *cagA* copies than WT mice-output colonies (Figure 1(d), 3(a)). Thus, a weaker immune response is associated with higher *cagA* copy number, while a stronger immune response is associated with lower *cagA* copy number. *H. pylori* colonies isolated from all groups except *Rag1*^−/−^^/−^ showed decrease in *cagA* copy number during infection, compared to average *cagA* copy number of input population. There were 11 strains isolated from 2 *Il10*^−/−/−^ mice that completely lost their *cagA*. In addition to an effect of host immune response toward a decrease in *cagA* copy number, a host-specific factor might affect this striking decrease of *cagA* in strains isolated from specific mice. It is known that the host immune response in mice exerts a driving force against *H. pylori* infection, and that strains colonizing robustly tend to have a less functional T4SS and to induce low IL-8 secretion.^59^ Although it has been reported that the recombination in *cagY* leads to the reduction of *H. pylori* virulence by lowering T4SS function,^53,59^ many other genes in which mutations affect virulence were also reported.^65^ Since CagA induces an immune response that is detrimental to bacterial survival,^38,39^ a decrease in *cagA* copy number might be a response of *H. pylori* to lower the host inflammatory response. Alternatively, decreased *cagA* copy might result from selection against strains expressing high *cagA* copy number. Our results do not permit us to distinguish between these two mechanistic explanations, but the latter seems more likely.

The positive correlation between *cagA* copy number and the virulence of laboratory-grown and mouse-output *H. pylori* strains has been shown in a previous study^62^ and this study (Figure 4, S2), respectively. In this study, we showed that the positive correlation between *cagA* copy number and the virulence of *H. pylori* strain was prominent among *cagY*-S colonies (Figure S2). Because CagA is translocated into host cells via the T4SS,^16,17,75^ it can be easily assumed that T4SS may also have an important role in regulating CagA-mediated host inflammatory response. It is notable that no or weak correlation between *cagA* copy number and virulence was observed in mice-output colonies at 8 weeks PI (Figure S3). This suggests that genetic changes related to T4SS function may occur during prolonged infection and result in a weakening of correlation between *cagA* copy number and the virulence of *H. pylori*.

Multiple linear regression analysis showed that IL-8 secretion of infected cells can be explained by *cagA* copy number and *cagY* recombination status better in *H. pylori* colonies isolated from *Il10*^−/−/−^ than from WT mice (Figure 5). This suggests that modulation of *H. pylori* virulence via *cagA* copy number and *cagY* recombination is more critical in a harsh host environment. However, recombination in *cagY* does not always lead to a loss of T4SS function, and *cagY*-mediated change of T4SS function may occur gradually rather than in an on-and-off fashion.^53,59^ Thus, even though binary modeling of *cagY* recombination status we used here provides a useful model to analyze changes in *cagA* copy number according to the host environment and strain capacity for IL-8 secretion, it should be interpreted cautiously.

Multiple linear regression analysis also showed that *cagA* and *cagY* play a role in an inflammatory response induced by *H. pylori* (Figure 5, Table 1). Our results imply that virulence of *H. pylori* might be regulated not only by a combined effect of change of *cagA* copy number and recombination in *cagY* but also by additional mechanisms that affect T4SS function. Many components of T4SS or other virulence factors of *H. pylori* have been reported to interact with CagA or affect T4SS function. For example, in addition to CagY, other T4SS components such as CagI, CagL, and CagQ were reported to interact with CagA,^69,70^ and to be indispensable for the formation of T4SS pilus.^76^ Hansen et al.^65^ also identified mutations in *cag*PAI genes including *cag5, cag10, cagA*, and *cagY* from mouse-passaged strains which lost T4SS function. On the other hand, outer membrane proteins such as OipA, BabA, and HopQ can affect CagA translocation and IL-8 secretion via T4SS.^66–68^ Thus, these proteins may also influence host inflammatory response by affecting T4SS function. Investigating genetic and phenotypic variations of other components of *H. pylori* and how those variations affect T4SS function and host inflammatory response can be an interesting topic for future study.

In conclusion, changes in *cagY* and in *cagA* copy number mediate *H. pylori* persistence in the face of the host inflammatory response. Whether these changes result from bacterial regulation or selective killing is unknown and remains a topic for further study.

## Materials and methods

### H. pylori *strains and culture conditions*

*H. pylori* strain PMSS1^77^ and its mouse-passaged derivatives acquired in a previous study^59^ were used in this study. A total of 339 mouse-passaged PMSS1 isolates recovered from 58 mice were examined for *cagA* copy number, *cagY* recombination, and capacity to induce IL-8 in AGS human gastric adenocarcinoma cells (ATCC #CRL-1739). Briefly, 10- to 12-week-old female C57BL/6 WT, *Rag1*^−/−/−^, or *Il10*^−/−/−^ mice were challenged with 2.5 × 10^9^ CFU of *H. pylori* PMSS1. All mice were purchased from The Jackson Laboratory. Stock numbers for C57BL/6 WT, *Rag1*^−/−^^/−^, or *Il10*^−/−^^/−^ mice were 000664, 002216, and 002251, respectively. Mice were euthanized at 4 or 8 weeks PI. Stomachs were taken and half of each stomach was homogenized, serially diluted, and plated onto brucella agar (BD BBL) plates supplemented with 5% heat-inactivated newborn calf serum (Invitrogen) and antibiotics (20 μg/ml amphotericin B, 200 μg/ml bacitracin, 3.3 μg/ml polymyxin B, 10.7 μg/ml nalidixic acid, and 100 μg/ml vancomycin).^53^ After the initial culture, brucella agar plates supplemented with 5% heat-inactivated newborn calf serum, 5 μg/ml trimethoprim, 10 μg/ml vancomycin, 2.5 units/ml polymyxin B, and 2.5 μg/ml amphotericin B were used for further passage.^53^ Cultures were incubated at 37°C under microaerophilic atmosphere (5% O_2_, 7.6% CO_2_, and 7.6% H_2_) generated by Anoxomat (Advanced Instruments), as previously described.^53^

### Southern blot analysis

To identify variation of *cagA* copy number in *H. pylori* population isolated from mice, Southern blot was performed as previously described.^61^ Briefly, *H. pylori* genomic DNA was digested with restriction enzyme SspI (New England Biolabs) for 2 h, then separated on a 0.5% agarose gel overnight at 0.75 V/cm. Separated DNA fragments were then transferred to a nylon membrane. Part of *cagA* gene of *H. pylori* strain PMSS1 was PCR-amplified using primers D008 and R008 (Table 2). The resulting 298-bp fragment was labeled with biotin using a North2South biotin Random Prime Labeling kit (Thermo Scientific) and used as a probe for Southern blot. Hybridization and detection were performed using North2South chemiluminescent hybridization and detection kit according to the manufacturer’s instructions. *cagA* copy numbers were determined based on fragment size and the known restriction map of the *cagA* locus.

**Table 2.** Primers used in this study

Primer name	Sequence (5ʹ → 3ʹ)	Reference
Generation of Southern blot probe		
D008	ATA ATG CTA AAT TAG ACA ACT TGA GCG A	61
R008	TTA GAA TAA TCA ACA AAC ATC ACG CCA T	
Amplification of *cagA* gene for qPCR		
RTcagAF	CCC TTA AAG GCT CGG TGA A	62
RTcagAR	TTT TCA AGG TCG CTT TTT GC	
Amplification of *ureA* gene for qPCR		
RTureAF	AAA AGC CGT TAG CGT GAA AGT	62
RTureAR	CCC GCT CGC AAT GTC TAA G	
Amplification of *cagY* gene for PCR-RFLP		
cagY:5157L24	CCG TTC ATG TTC CAT ACA TCT TTG	59
cagX:1515U22	CTA TGG TGA ATT GGA GCG TGT G	

To determine the *cagA* copy number of *H. pylori* population, Southern blot profiles were densitometrically analyzed using ImageJ software (National Institute of Health, USA). Density of individual Southern blot bands corresponding 4, 3, 2, and 1 *cagA* copy was respectively measured, and these values were called ‘raw density’. Since an isolate carrying multiple *cagA* genes will have multiple probe-binding sites, raw densities were divided with *cagA* copy number of each band from which the raw density was measured, and these values were called ‘calibrated density’. Then, the sum of all raw densities of a population was divided with the sum of all calibrated densities of the population to get an average *cagA* copy number of the *H. pylori* population. This process is summarized in Figure S4.

### Quantitative polymerase chain reaction (qPCR)

*cagA* copy number of each mouse output colony was measured by qPCR using the 2^−ΔΔCT^ method.^78,79^ *ureA* was used as a reference gene and *H. pylori* strain PMSS1/*cagA*-S^F^-1,^62^ which has a single copy of *cagA*, was used as a calibrator. Briefly, −ΔΔC_T_ can be obtained by calculating ΔC_T_ of target − ΔC_T_ of calibrator, where ΔC_T_ = C_T_ of *cagA* − ΔC_T_ of *ureA*, and C_T_ means a threshold cycle. Thus, 2^−ΔΔCT^ of a certain single colony isolate is a multiple of *cagA* copy number compared to that of calibrator strain, so it can be regarded as a *cagA* copy number of a certain single colony isolate. Primers used for amplification of *cagA* or *ureA* are described in Table 2. Genomic DNA was isolated from each *H. pylori* strain using Nucleospin 96 Tissue kit (Machery-Nagel) under the manufacturer’s instruction. qPCR reaction mixtures contained 1 ng of template DNA, 10 μl of SYBR Premix *Ex Taq* (Tli RNaseH Plus; Takara Bio), 0.4 μl of ROX reference dye, and 200 nM of each primer, in a total reaction volume of 20 μl. Reaction mixtures were transferred into a MicroAmp optical 96-well plate (Applied Biosystems), which was sealed with MicroAmp optical adhesive film (Applied Biosystems). qPCR was performed as follows: 95°C for 30 s, 40 cycles of 95°C for 5 s and 60°C for 30 s. After the amplification was finished, melting curve was analyzed to ensure proper annealing of primers and amplification of target sequence. QuantStudio 6 Flex Real-Time PCR System (ThermoFisher Scientific) was used to perform qPCR and data were analyzed using QuantStudio Real-Time PCR Software v1.0. All samples were run in triplicate for each primer pair. Results obtained from different qPCR runs were calibrated as previously described.^80^ Mean *cagA* copy numbers were presented with standard deviation in parentheses.

### cagY *PCR-restriction fragment length polymorphism (RFLP)*

Genomic DNA isolated from each mouse output colony was subjected to PCR-RFLP to detect the recombination in *cagY*, as described previously.^53^ The region containing *cagY* was amplified using a previously described primer pair (Table 2) with Expand Long Template PCR System (Roche Diagnostics). PCR reaction mixtures contained 100 ng of genomic DNA, 0.3 μM of each primer, 0.35 mM of each dNTP, 3.75 U of Expand *Taq* DNA polymerase, and 1× buffer containing 1.75 mM MgCl_2_ in total volume of 50 μl. PCR products were purified using QIAquick PCR purification kit (QIAGEN Sciences) and 840 μg of each purified DNA was separately digested overnight with DdeI or BfucI (New England Biolabs) at 37°C. After digestion, DNA was electrophoresed in a 5% agarose gel and stained with ethidium bromide. Size of the amplicon and two RFLP patterns of each mouse output colony were compared with those of the PMSS1 input strain. If *cagY* from an output colony differed in size or RFLP pattern from that of PMSS1, it was considered to have undergone *cagY* recombination.

### IL-8 enzyme-linked immunosorbent assay (ELISA)

IL-8 secretion from AGS cells was measured by ELISA as previously described.^81^ AGS cells were maintained in RPMI 1640 (Gibco BRL) supplemented with 10% FBS (Invitrogen) and 1% penicillin/streptomycin (Invitrogen). For IL-8 ELISA, AGS cells were seeded on a 6-well cell culture plate with a density of 5 × 10^5^ cells/well in 1.8 ml of RPMI 1640 with 10% FBS, and then incubated 24 h at 37°C. *H. pylori* strains were diluted in 200 μl of brucella broth (BD BBL) and added to AGS cells with a multiplicity of infection of 100. *H. pylori* strain PMSS1 and its isogenic Δ*cagY* mutant^53^ were used as positive and negative controls, respectively. IL-8 induction of output colonies was expressed as a relative unit compared to that induced by PMSS1.

### Statistical analysis

Statistical analyses were conducted using IBM SPSS version 23.0 software (IBM, Armonk, NY), and R version 3.5.3 (R core team) with packages ggplot2 (https://ggplot2.tidyverse.org/), ggbeeswarm (https://CRAN.R-project.org/package=ggbeeswarm), and plot3D (https://CRAN.R-project.org/package=plot3D). Intraclass correlation coefficient (ICC, two-way mixed, single measure) was used to examine agreement between *cagA* copy number measured by densitometry analysis of Southern blot and by qPCR. Average *cagA* copy number of mouse output colonies was compared to that of the input PMSS1 population using a one-sample *t* test and compared to each other using a two-sample *t* test. Homogeneity of variances across each group was analyzed by Levene’s test. Correlation between *cagA* copy number and relative IL-8 induction was measured by calculating Pearson correlation coefficient. Multiple linear regression was calculated based on *cagA* copy number and *cagY* recombination status. Adjusted R^2^ values were used to evaluate the explanatory power of regression models. All analyses were considered to be statistically significant when *P* value is less than 0.05.

## Data Availability

All data are contained in the article and the supporting information.
